# Molecular Characterization and Expression of a Heat Shock Protein Gene (HSP90) from the Carmine Spider Mite, *Tetranychus cinnabarinus* (Boisduval)

**DOI:** 10.1673/031.010.11201

**Published:** 2010-07-15

**Authors:** Hongzu Feng, Lan Wang, Yinghong Liu, Lin He, Ming Li, Wencai Lu, Chuanhua Xue

**Affiliations:** ^1^ Chongqing Key Laboratory of Entomology and Pest Control Engineering, College of Plant Protection, Southwest University, Chongqing 400716, China; ^2^ Plant Science College, Tarim University, Ala, Xingjiang 843300, China

**Keywords:** abamectin, comparative quantitative expression, gene cloning, temperature shock

## Abstract

In this study, the cDNA of *Tetranychus cinnabarinus* (Boisduval) (Acarina: Tetranychidae) HSP90 (designated TcHSP90) was cloned using a combination of the homology cloning and rapid amplification of cDNA ends (RACE) approaches. The full-length cDNA of TcHSP90 is 2595 bp, including a 5′-untranslated region (UTR) of 177 bp, 3′-UTR of 249 bp, and an open reading frame (ORF) of 2169 bp. The ORF encodes a polypeptide of 722 amino acids with a predicted molecular weight of 83.45 kDa and a theoretical isoelectric point of 4.81. There is an mRNA polyadenylation signal of ATTAAA at the positions 2558–2564. In addition, the expression pattern of TcHSP90 mRNA relative to that of β-actin gene in the three stains of *T. cinnabarinus* (AbR, abamectin-resistant strain; HR, heat-resistant strain; SS, the susceptible strain) were examined by using fluorescent real time quantitative PCR after the impact of abamectin, high and low temperature, respectively. The results showed that under the normal condition, the mRNA level of TcHSP90 was 1.64 and 1.29-fold higher in the AbR and HR than in SS, respectively. After 8 h treatment with abamectin, the TcHSP90 mRNA levels of SS, AbR, and HR were 1.25, 1.87, and 2.05-fold higher than those of their untreated controls, respectively. The TcHSP90 mRNA levels of SS, AbR, and HR were also significantly increased after being induced at 40° C for 1 h, and they were 3.76, 3.42, and 3.79-fold higher than those of their untreated controls, respectively. The mRNA level of TcHSP90 was also significantly increased after being induced at 4° C for 1 h. These results suggest that TcHSP90 might be involved in the abamectin and extreme temperature resistance or tolerance.

## Introduction

Under high temperature or other adverse environments, many organisms can be induced to synthesize a new set of proteins, designated as the heat shock proteins (HSPs) (Tissieres et al. 1974). The HSPs are divided into four families according to their molecular weights (Morimoto et al. 1993), including the HSP90 family (83 ∼ 95 kDa), the HSP70 family (66∼78 kDa), the HSP60 family, and the smHSP family (small heat shock protein). Among these families, HSP70 and HSP90 are the most universal HSPs (Parsell and Lindquist 1994). Previous studies showed that HSPs, especially HSP70 and HSP90, possessed important functions in high-temperature resistance. Also, the acquiring rate of heat resistance showed a positive correlation with the accumulation rate of HSPs; the decrease of heat resistance and the degradation of HSPs evolved coordinately (Denlinger et al. 1998; Sörensen et al. 2001; Chen 2005). Unlike other HSPs, HSP90 accounts for 1–2% of all the cellular proteins in non-stress cells. Some studies show that HSP90 promotes the refolding of denatured proteins in heat or other stress conditions and degrades the protein ions (Caplan 1999; Pearl et al. 2000). A recent study shows that HSP90 is involved in many physiological functions (Christine et al. 2002).

It is known that HSPs can be induced not only by heat, but also by other factors, such as environmental pollution, pesticides, heavy metals, UV radiation, acids, antibiotics, and hormones (Anderson et al. 1983; Lee and Dewey 1988; Koga and Takumi 1995; Hartke et al. 1995; Patil et al. 1996). Interestingly, a moderate heat shock may confer *Sarcophaga crassipalpis* (Chen et al. 1987) and *Drosophila melanogaster* (Bueton et al. 1988) an ability to tolerate a relatively low lethal temperature. In another study (Patil et al. 1996), the sub-lethal concentration of propoxur induced the resistance of *Anopheles stephensi* and *Aedes aegypti* to a higher temperature. On the other hand, exposure to high temperature for several hours can protect the insect from the pesticide (Patil et al. 1996). All these observations suggest that an organism can survive under the cross-protection of different stresses: one type of resistance can be induced by another type.

Due to its strong reproductive ability, short generation time, small movement area, high inbreeding rate and frequent insecticide-contacted opportunity, the problem of resistance to acaricides is more severe for *Tetranychus cinnabarinus* (Boisduval) (Acarina: Tetranychidae) than for other crop insect pests. *T*. *cinnabarinus* is widely distributed in China and is difficult to prevent or control, which has caused severe damage to cotton and vegetable crops. The abamectin used in production has become the leading medicament to control insect and mite pests because of its unique mechanisms and excellent control effect. However, pest resistance to abamectin has rapidly developed from its large-area and high-reliance use.

He et al. (2005) showed that the fecundity of the abamectin-susceptible strain had a higher fecundity than that of the abamectin-resistant strain within the temperature range of 20–28° C, while the amount of oospheres produced by the resistant strain was greater at 34° C than that of the sensitive strain (He et al. 2005), suggesting that the abamectin-resistant strain has a reproductive advantage and better adaptability at a higher temperature. Thus, it is necessary and important to elucidate the mechanism of high temperature fitness generated by *T. cinnabarinus*, which achieves the resistance to insecticides prior to treatment with higher temperature. The efforts to study this intriguing phenomenon will help us to better understand the resistant fitness of mites and other insects under environmental stress. In this study, the full length cDNA of TcHSP90 was cloned and sequenced using RT-PCR and RACE techniques. The expression of the TcHSP90 gene in abamectin susceptible and resistant strains, as well as in a high-temperature tolerant strain, were also quantitatively investigated under the treatments of abamectin, cold, and heat using real-time fluorescence quantitative PCR. The objective of the study was to help to establish a theoretical basis for the mechanism of high-temperature fitness in abamectin-resistant *T. cinnabarinus.*

## Methods and Materials

### Mites

A colony of *T. cinnabarinus* was established from the mites collected from the field of Beibei, Chongqing, China, which were reared under pesticide-free conditions (26° C and 70% RH). About 1200 mites were collected from cowpea and were regarded as the original colony. This colony was never exposed to acaricides and considered as the SS.

### Resistance selection and bioassay

The resistant strain of *T. cinnabarinus* was selected from the susceptible strain. Acaricide selection was carried out by spraying abamectin with Yangtze-08, and the selective pressure was maintained at about 70% of the population mortality rate. The surviving *T. cinnabarinus* were transferred to new cowpea seedlings 24 h after spraying, and the mortality rate was recorded at that time.

Surviving *T. cinnabarinus* were allowed to lay eggs on the new cowpea seedlings for 1–2 days. Then, the survivors were removed until the eggs of the same generation grew to adult, and abamectin was sprayed again. After each treatment, the survivors were cultured and selected again at the next generation. The bioassay procedures generally followed the recommended methods of Food and Agriculture Organization. After 48-generation continuous selection, the resistant fold was up to 11.05, and the colony was defined as the abamectin-resistant strain.

The high-temperature-resistant strain was also isolated from the susceptible strain. The mites were reared in a thermal incubator at 34° C, 75–80% RH and a photoperiod of 14:10 L:D. The incubator temperature was raised 1° C after every two generations. The final temperature was stabilized at 40° C, and this strain was defined as the high-temperature-resistant strain.

### RNA extraction and cDNA synthesis

Total RNA was extracted from the adult female spider mites of *T. cinnabarinus* (≈ 20 mg) with TRIzol (Invitrogen, www.invitrogen.com) according to the manufacturer's instructions. The RNA quality was assessed by electrophoresis on 1.2% agarose gel. Total RNA was treated with RQ1 RNase-Free DNase (Promega, www.promega.com) to remove contaminating DNA from the total RNA, and cDNA was synthesized from 2 µg total RNA by M-MLV reverse transcriptase (Promega) following the manufacturer's protocol with the oligo (dT) primer 5′-GGCCACGCGTCGACTAGTAC (T)16(A/C/G)-3′.

### Cloning and sequencing of TcHSP90

Two degenerate primers S2: 5′-GTNTTYAT HATGGAYAAYTG-3′ and A2: 5′-ACRTAY TCRTCDATNGGYTC-3′ were designed based on the conserved sequence of known HSP90s to amplify the partial fragment of TcHSP90 gene from *T*. *cinnabarinus.* The PCR reaction was performed in a 25 µl reaction volume containing 2.5 µl of 10×PCR buffer, 1.6 µl of MgCl2 (25 m*M*), 2 µl of dNTP (2.5 m*M*), *2* µl of each primer (10 mM), 13.5 µl of PCR-grade water, 0.4 µl (1 U) of Taq polymerase (Promega) and 1 µl of cDNA template. The PCR temperature profile was 94° C for 3 min followed by 34 cycles of 94° C for 1 min, 54° C for 30 s, 72° C for 30 s and a final extension step at 72° C for 10 min. The PCR products were separated on 1.2% agarose gel and purified by the PCR fragment purification kit (TaKaRa, www.takarabio.com). The purified PCR product was ligated into the pMD18-T vector (TaKaRa) and transformed into competent *Escherichia coli* cells. The recombinants were identified through the blue-white color selection in ampicillin-containing LB plates and screened with both forward and reverse primers. Fourteen positive clones were selected for sequencing (Invitrogen), and the resulting sequences were verified and subjected to further clustering analysis.

The 5′ end of TcHSP90 cDNA was obtained by the RACE technique. Two specific reverse primers, 5GSP1: 5′-GCAGCAACTTGTTCCT TAGATTCACC-3′ and 5GSP2: 5′-GCTTCCTTATCC TCGGCTACTTCTTCG-3′ were designed based on the partial sequence amplified by the degenerate primers. The PCR amplification was performed with the same reaction system as described before with the adaptor primers (UPM) and the gene-specific primer (5GSP1) by the 5′ RACE system (Invitrogen), and then a nested PCR was carried out with adaptor primers (NUP) and specific primers (5GSP2).

The 3′ end of TcHSP90 was amplified with sense primer 3GSP 5′-GAAGTAGCCGAGGATAAGGAAGC-3′ and adaptor primers 5′-CTGATCTAGAGGTACCGGATCC-3′ with 1 µl of cDNA template. The full-length sequence was verified by sequencing the fragment amplified by the primers F4: 5′-TGTTCATCTCACATTTCCACAC-3′ and R4: 5′-AACTACTTTCTATCCCA TCCCTT-3′ (located at 5′ UTR and 3′ UTR of TcHSP90).

### Bioinformatic analysis of the target gene

The sequence similarity search at both nucleotide and amino acid levels were performed with the BLAST program at the National Center for Biotechnology Information (http://www.ncbi. nlm.nih.gov/BLAST/). The inferred amino acid sequence was analyzed with the Expert Protein Analysis System (http://www.expasy.org/). Multiple alignment of TcHSP90 was performed with the Clustal W Multiple Alignment program (http://www.ebi.ac.uk/clustalw/). A phylogenic tree was constructed by CLUSTAL (version X1.83) (Thompson et al. 1997) and MEGA (version 3.1) (Kumar et al. 2004) based on the obtained TcHSP90 sequences and other known HSP90 sequences. The bootstrap analysis was used with 1000 replicates to estimate the confidence of the branches produced by the neighbor-joining analysis.

### Quantitative analysis of the TcHSP90 mRNA expression

Cowpea seedlings with *T*. *cinnabarinus* SS strain were treated with the following method: for heat and cold shock experiments, six treatments (200 adult female *T*. *cinnabarinus* per treatment) were transferred to lightconstant temperature incubator exposed to 4° C, 7° C, 10° C, 34° C, 37° C, and 40° C for 1 h and abamectin treatment (100,000 × 8 h), respectively. The AbR and HR were treated with cold shock (4° C, 1 h), heat shock (40° C, 1 h), and abamectin treatment (100,000 × 8 h), respectively. After the *T. cinnabarinus* recovered for 30 min, total RNA of each treatment and the control sample (26° C) was immediately extracted from whole bodies using Trizol reagent (Invitrogen) according to the manufacturer's protocol. In all cases, total RNA was treated with DNase I (TaKaRa). Concentration of total RNA was determined by measuring ultraviolet (UV) absorbance at 260 nm. RNA purity was checked by determining the *A*260/*A*280 ratio, and its integrity was checked by formaldehyde agarose gel electrophoresis. All RNAs were stored at -80° C until used.

Real-time quantitative PCR was performed on Mx3000P Florescent Real-time Quantitative PCR (Stratagene) with β-actin (GenBank accession number: ABV82698) gene from *T*. *cinnabarinus* as a reference. Expression of β-actin showed no response to temperature (*n* = 3 individuals) in preliminary experiment (data not shown). The efficiency of PCR amplification for gene specific primers was analyzed by one cDNA sample with five serial dilutions and three technical replications. The PCR mixture contained 10 µl, 2× SYBR Green Mix, 0.8 µl primers, respectively, 2.0 µl cDNA and 6.4 µl ddH2O. The primers used for TcHSP90 were 5′-TCCACAACGTCATTCCTCTCGCAT-3′ and 5′-TCCAGAG GAGGCATTTCAGCT TCA-3′ with a product of 117 bp; and the primers for β-actin were: 5′-CAGCCATGTATGTTGCCATC-3′ and 5′-AAATCACGACCAGCCAAATC-3′ with a product of 166 bp. The PCR condition was performed as follows: 95° C for 3 min, followed by 40 cycles at 95° C for 30 s, 60° C for 30 s and 68° C for 30 s. A melting curve program was run immediately after the PCR program, and the data were analyzed with automatic software. The threshold cycle (Ct) values were used to quantify the target gene expression for each sample. The amplification folds of TcHSP90 in SS, AbR and HR were calculated with the 2-ΔΔCt method (Livak et al. 2001). The real-time PCR analysis was independently repeated three times for each sample.

## Results

### Cloning and sequencing of the TcHSP90 gene

The PCR product amplified by the degenerate primers was 485 bp long, and its nucleotide sequence was homogeneous to other known HSP90s. TcHSP90-specific primers R2, R3, and F2 were designed based on the above sequences, and used for the full-length cDNA cloning. With RACE and nested PCR approaches, two fragments corresponding to the 5′ and 3′ end of the TcHSP90 cDNA were amplified. Finally, a nucleotide sequence of 2595 bp representing the complete cDNA sequence of TcHSP90 was obtained by assembling the above fragments.

### Characterization of TcHSP90

**Figure 1.**
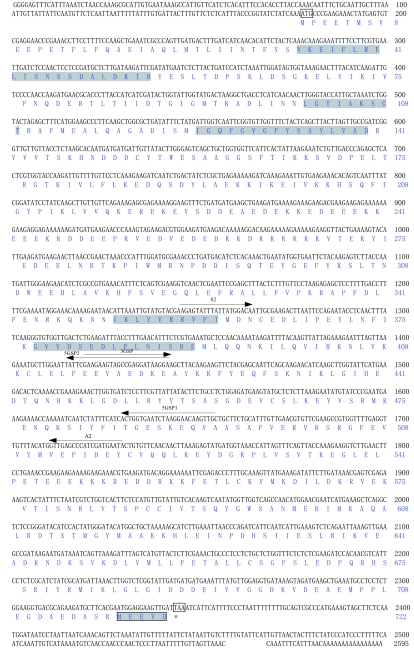
Nucleotide and deduced amino acid encoding region of **TcHSP90** (including 5′ and 3′ UERs). Signature sequences of Hsp90 family and C-terminal five amino acids(MEEVD) are shown in shaded parts, start codon and stop codon are boxed. The tailing signals are shown in bold. Arrows above the nucleotide sequences represent the position of the different synthetic primers (degenerate primers and Specific primers for RACE experiments) used in PCR. High quality figures are available online.

The cDNA sequence of TcHSP90 was deposited in GenBank (accession no. EU851046). The full-length cDNA of TcHSP90 is 2595 bp long, including a 5′-untranslated region (UTR) of 177 bp, a 3′-UTR of 249 bp, a canonical polyadenylation signal sequence ATTAAA, a poly (A) tail, and an open reading frame (ORF) of 2169 bp. The ORF encodes a polypeptide of 722 amino acids, whose predicted molecular weight is 83.45 kDa and whose theoretical isoelectric point is 4.81. The protein contains the five amino acid blocks defining the HSP90 protein family (NKEIFLRELISN[S/A]SDALDKIR, LGTIA[K/R]SGT, IGQFGVGFYSA[Y/F] LVA[E/D], IKLYVRRVFI, GVVDS[E/D] DLPL N[I/V]SRE) as well as a consensus sequence MEEVD at the C-terminus that are highly conserved in the TcHSP90 sequence (Figure 1). The SMART program analysis revealed a typical histidine kinase-like ATPases domain at the positions 34-188, which is ubiquitous in all HSP90 family members.

### Homology analysis of TcHSP90

**Figure 2.**
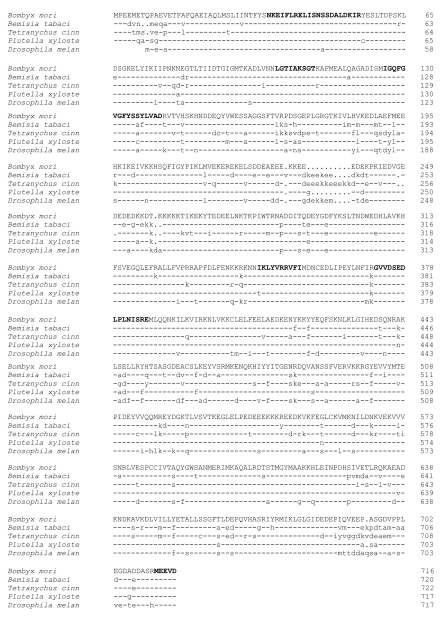
Comparison of the amino acid sequences of the HSP90 protein family. The GenBank accession numbers and species names were as follows: *Bemisia tabaci*: AAZ17403, *Bombyx mori:* BAB41209, *Drosophila melanogaster.* NP_523899, *Plutella xylostella:* BAE48742, *Tetranychus cinnabarinus:* ACF75907. High quality figures are available online.

Multiple alignments of TcHSP90 with that of other species showed high conservation (Figure 2). The BLAST results of the deduced amino acid sequence of TcHSP90 showed that the sequence obtained was closer to arthropods than to other organisms. The five HSP90 signature sequences (Gupta 1995), were observed in each of the HSP90 proteins compared (Figure 2, bold). Relative to the canonical signature sequences, somewhat fewer substitutions were observed in the signature sequences of the arthropods. Among the HSP90 proteins compared, the highest percent identity of *T. cinnabarinus* HSP90 was with HSP90 from *Bombyx mori* (79.97% identical). The lowest percent identity and similarity was with HSP90s from *D. melanogaster* (77.09% identity). Identity of HSP90 was high within the arthropods, especially in the signature regions of the HSP90 family.

**Figure 3.**
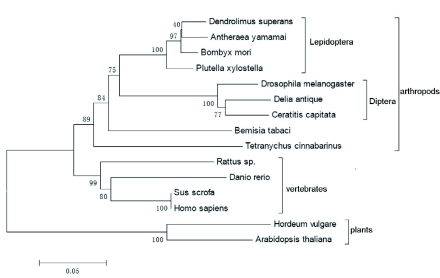
A phylogenetic tree of HSP90 family members constructed with the neighbor-joining method. Numbers at each branch indicate the percentage of times a node is supported in 1000 boostraps pseudoreplication by neighbour-joining. the species names were as follows: *Antheraea yamamai*: BAD15163, *Arabidopsis thaliana:* BAA00615, *Bemisia tabaci*: AAZ17403, *Bombyx mori:* BAB41209, *Ceratitis capitata:* CAJ28987, *Danio rerio:* NP_571403, *Delia antique:* CA164494, *Dendrolimus superans:* ABM89112, *Drosophila melanogaster:* NP_523899, *Homo sapiens:* NP_005339, *Hordeum vulgare:* AAP87284, *Plutella xylostella:* BAE48742, *Rattus sp.:* AAB23369, *Sus scrofa:* NP_999138, *Tetranychus cinnabarinus:* ACF75907. High quality figures are available online.

Based on the sequences of HSP90s, a phylogenetic tree was constructed with CLUSTAL X1.83 (Thompson 1997) and MEGA 3.1. As shown in Figure 3, plant and animal HSP90s are separated and form two distinct branches in the tree. In the branch of animals, vertebrates and arthropods are separated and form two distinct branches in the tree. All insects are clustered together and form a sister group to the branch of mites. The relationships displayed in the phylogenic tree are in concurrence with traditional taxonomy.

### Quantitative analysis of TcHSP90 gene expression

Fluorescent real-time quantitative PCR was used to measure the mRNA expression level of TcHSP90 in *T. cinnabarinus* with the treatments of abamectin, high temperature, and low temperature. When Ct values were used to generate a log-linear regression plot, the standard curve for the house keeping gene β-actin showed a strong relationship (r2= 0.9991; PCR efficiency = 99.5%). The efficiency of PCR amplification for each target gene of TcHSP90 tested showed that the amplification efficiencies were 96.2% for TcHSP90 primers. The amplification efficiencies experiments indicated that each pair of primer of β-actin and TcHSP90 used for real-time PCR did not violate assumptions of the two ddCt method. Melting curve analysis and gel electrophoresis showed only target gene was synthesized.

**Figure 4.**
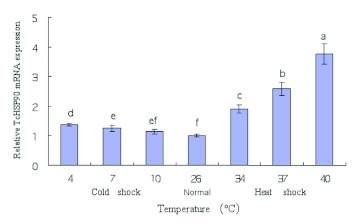
Comparative quantitative RT-PCR analysis of the relative expression of TcHSP90 in SS at different temperatures. Different letters above each bar indicate statistical difference by ANOVA analysis followed by the Duncan's Multiple Comparison test (p<0.05). High quality figures are available online.

**Figure 5.**
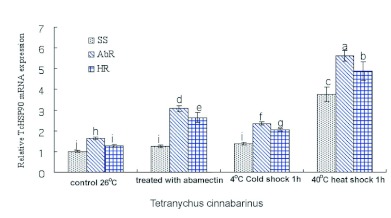
Comparative quantitative RT-PCR analysis of the relative expression of TcHSP90 after abamectin, cold and heat shock treatment. Different letters above each bar indicate statistical difference by ANOVA analysis followed by the Duncan's Multiple Comparison test (p<0.05). High quality figures are available online.

The mRNA expression level of TcHSP90 in SS showed the tendency of rising after heat and cold shock, but the expression level after heat shock was higher than that after cold shock (Figure 4). Under the normal condition, the mRNA expression levels of TcHSP90 in AbR and HR were 1.64-fold and 1.29-fold higher, respectively, than that in SS. Compared with the corresponding control groups, the TcHSP90 mRNA level was increased at 8 h after the abamectin treatment, which was 1.25-fold higher in SS, 1.87-fold higher in AbR, and 2.05-fold higher in HR (Figure 5). After being induced by high and low temperature, the TcHSP90 mRNA level also increased. Specifically, after being induced at 40° C for 1 h, the mRNA levels of SS, AbR, and HR were 3.76, 3.42 and 3.79-fold higher than their corresponding control groups, respectively, all of which were significant (p < 0.05); after being induced at 4° C for 1 h, the mRNA levels of SS, AbR, and HR were 1.38, 2.36, and 2.06-fold higher than their corresponding control groups, respectively, all of which were also significant (p < 0.05).

## Discussion

In this study, complete cDNA sequence of HSP90 gene from *T. cinnabarinus* was reported. Conserved sequences and characteristic motifs, such as HSP90 family signatures, and histidine kinase (from 35 to 188 amino acid residues) (Gupta 1995) were found, as well as the major structural and functional domains typically in HSP90 (Buchner 1999; Caplan 1999) in the inferred TcHSP90 protein sequence. The C-terminal EEVD motif of TcHSP90 is the cytoplasmic localization signal of the heat-shock proteinfamily, which is recognized by the TPR domains of HOP (HSP70 and HSP90 organizing protein) (Scheufler 2000) and involved in assembling the multiple molecular chaperone. In addition, the presence of motif MEEVD at the C-terminus is a character shared among all the eukaryotic HSP90 proteins. The sequence similarity analysis revealed that the inferred amino acid sequence of TcHSP90 shared high similarities with other known HSP90s, especially with those from insects of arthropods. Based on sequence alignment, structure comparison and phylogenetic analysis, we concluded that TcHSP90 was a cytosolic member of HSP90 family. Studying the divergence of TcHSP90 from *T. cinnabarinus* has important theoretical and practical significance in terms of how to study environmental stress response.

In many species, sub-lethal temperatures can often induce the greatest amounts of HSP mRNA (Feder and Hofmann 1999). The temperatures 0° C and 40° C are sub-lethal for locust eggs (Jing and Kang 2003) and can induce an increase in HSPs expression (Li et al. 2000). HSPs are generally expressed at low levels under non-stress conditions, but increase rapidly in response to a stress (Sonoda et al. 2006).

The mRNA expression of TcHSP90 in abamectin and high and low temperature stress conditions was studied based on fluorescent real time quantitative PCR. The results showed that standard expression levels of TcHSP90 mRNA was higher in AbR and HR than in SS, suggesting that a higher amount of HSP90 in *T. cinnabarinus* may be helpful for adaptation to temperature and abamectin stress. The TcHSP90 mRNA in AbR and HR was higher than in SS given the treatments of abamectin, high temperature, and low temperature. This observation may be ascribed to physiological functions of cross protection in *T. cinnabarinus* under different stress conditions. Greater accumulation of HSP90 in *T. cinnabarinus* induced by insecticide resistance and high temperature resistance protected the organism from stress injuries. This may provide a theoretical basis for explaining the fitness advantage of resistance of insects or mites to abamectin.

While HSP70 has been widely accepted and applied as a biomarker of unhealthy environmental factors, studies about HSP90 are rare (Arts et al. 2004; Hallare et al. 2005; Soon et al. 2006). The results of this study showed that TcHSP90 had protection functions not only in high-stress temperatures, but also under stresses from an insecticide. Therefore, HSP90 is a potential biomarker that can detect environmental factors of stress to the organism. As far as this notion is concerned, some studies have been carried out in soil and marine organisms (Köhler et al. 1998,^ 2001^; Arts et al. 2004; Hallare et al. 2005; Soon et al. 2006). However, it should be noted that a single biomarker cannot reflect the overall physiological state of an organism. More and more attention should be paid to the comprehensive functional evaluation using various biomarkers. This study showed that TcHSP90 was susceptible to the stress responses induced by abamectin and high temperature, and further efforts are needed to explore the response to environmental effects using HSP90 and HSP70 together.

There is a significant body of evidence that shows that animal cells have evolved a variety of elaborate defense and repair mechanisms to protect them against the negative effects of natural environmental stressors (Pruski & Dixon 2007). Cloning and characterization of the TcHSP90 provides a tool for investigating stress-related responses in *T*. *cinnabarinus.* Furthermore, the results of our study could help determine specific techniques to manage the acarid group which includes many agricultural pests and disease vectors.
